# A Microbe Associated with Sleep Revealed by a Novel Systems Genetic Analysis of the Microbiome in Collaborative Cross Mice

**DOI:** 10.1534/genetics.119.303013

**Published:** 2020-01-02

**Authors:** Jason A. Bubier, Vivek M. Philip, Christopher Quince, James Campbell, Yanjiao Zhou, Tatiana Vishnivetskaya, Suman Duvvuru, Rachel Hageman Blair, Juliet Ndukum, Kevin D. Donohue, Carmen M. Foster, David J. Mellert, George Weinstock, Cymbeline T. Culiat, Bruce F. O’Hara, Anthony V. Palumbo, Mircea Podar, Elissa J. Chesler

**Affiliations:** * The Jackson Laboratory, Bar Harbor, Maine 04609; † Genome Science and Technology Program, University of Tennessee, Tennessee 37830; ‡ Biosciences Division, Oak Ridge National Laboratory, Tennessee 37830; § School of Engineering, University of Glasgow, G12 8LT, United Kingdom; ** Department of Natural Sciences, Northwest Missouri State University, Maryville, Missouri 64468; †† Department of Biostatistics, State University of New York at Buffalo, New York, 14260; ‡‡ Signal Solutions, LLC, Lexington, Kentucky 40506; §§ Electrical and Computer Engineering Department, University of Kentucky, Lexington, Kentucky 40508; *** Department of Biology, University of Kentucky, Lexington, Kentucky 40508

**Keywords:** sleep, genetics, genomics, bioinformatics, behavior

## Abstract

The microbiome influences health and disease through complex networks of host genetics, genomics, microbes, and environment. Identifying the mechanisms of these interactions has remained challenging. Systems genetics in laboratory mice (*Mus musculus*) enables data-driven discovery of biological network components and mechanisms of host–microbial interactions underlying disease phenotypes. To examine the interplay among the whole host genome, transcriptome, and microbiome, we mapped QTL and correlated the abundance of cecal messenger RNA, luminal microflora, physiology, and behavior in a highly diverse Collaborative Cross breeding population. One such relationship, regulated by a variant on chromosome 7, was the association of *Odoribacter* (Bacteroidales) abundance and sleep phenotypes. In a test of this association in the BKS.Cg-*Dock7^m^* +/+ *Lepr^db^/*J mouse model of obesity and diabetes, known to have abnormal sleep and colonization by *Odoribacter*, treatment with antibiotics altered sleep in a genotype-dependent fashion. The many other relationships extracted from this study can be used to interrogate other diseases, microbes, and mechanisms.

## Abstract (precis)

Host genetic diversity provides a variable selection environment and physiological context for microbiota and their interaction with host physiology. Using a highly diverse mouse population, Bubier et al. identified that Odoribacter abundance influences sleep archi-tecture in a manner...

THE human microbiome has been implicated as an important factor in health and disease (Wen *et al.* 2008); however, the mechanisms by which it influences human physiology are largely unknown. Experiments that manipulate specific genetic, molecular, and microbial components of the microbe–host interface are essential for the dissection of these mechanisms (Vijay-Kumar *et al.* 2010), but the identification of targets for experimental manipulation remains a significant challenge. However, both microbial community composition and its effects on host health are modulated by host characteristics that exhibit heritable variation (Benson *et al.* 2010; Campbell *et al.* 2012; McKnite *et al.* 2012; Snijders *et al.* 2016), providing the opportunity to use population systems genetic strategies to identify genetic variants and associated traits to serve as entry points for investigating key functional pathways at the microbe–host interface (Willing *et al.* 2010; McKnite *et al.* 2012; Knights *et al.* 2014).

Studies of the gut microbiome have produced convincing evidence for a microbial influence over many host traits including human gastrointestinal disorders (Willing *et al.* 2010; Knights *et al.* 2014; Machiels *et al.* 2014), metabolic traits, diabetes (Wen *et al.* 2008; Vijay-Kumar *et al.* 2010), and obesity (McKnite *et al.* 2012; Carlisle *et al.* 2013; Parks *et al.* 2013). Perhaps more surprising is the influence of gut microbiota and the metabolites they produce on the brain (Bravo *et al.* 2011; Lewin *et al.* 2011; Carter 2013; Valles-Colomer *et al.* 2019) and circadian behaviors such as sleep (Leone *et al.* 2015). Despite the importance of these microbial influences, the mechanisms of many of these interactions remain unknown.

There have been many well-documented relationships between host genetic variation, intestinal flora composition, and disease reported in human genetic analyses (Deloris Alexander *et al.* 2006; Khachatryan *et al.* 2008; Turnbaugh *et al.* 2009; Goodrich *et al.* 2014; Jacobs and Braun 2014; Knights *et al.* 2014). Because mice and humans harbor similar microbiota at high taxonomic levels (Ley *et al.* 2008; Krych *et al.* 2013), systems genetic analysis in laboratory mice can be an effective tool for discovering the mechanisms of host–microbe interactions in a large-scale, data-driven manner. This quantitative genetic approach provides a means of holistic assessment of the relationships between hosts, microbes, and diseases through the use of population genetic variation, one of the greatest determinants of microbial community composition in mice (Deloris Alexander *et al.* 2006; Campbell *et al.* 2012). The study of natural genetic variation (Campbell *et al.* 2012) and engineered mutations (Turnbaugh *et al.* 2006; Spor *et al.* 2011) also enables deep dissection of the biology of the microbiome, and discovery of host genetic loci that regulate microbial abundance (Benson *et al.* 2010; McKnite *et al.* 2012). The transcriptome of the cecum provides insight into the host microenvironment by quantifying the relative abundances of transcripts encoding host pathways involved in metabolic responses, the production and presentation of cell-surface antigens, and constituents of the immune system, such as the gut-associated lymphoid tissue, among other host processes that both shape and respond to gut microbiota.

The Collaborative Cross (CC) mouse population, (Churchill *et al.* 2004; Chesler *et al.* 2008) constructed from the cross of eight diverse inbred progenitor strains, was designed for high-precision (Philip *et al.* 2011) and high-diversity systems genetic analysis. The host genetic variation among this population results in diverse microbiome compositions (Campbell *et al.* 2012), and physiological and behavioral phenotypes. This population has been used to identify QTL regulating the abundance of fecal microbes (Snijders *et al.* 2016). Here, we use host transcriptomic, in addition to genetics and microbiome, analysis to find host mechanisms related to disease. Genetic correlations among these characteristics are used to construct systems genetic networks (Figure 1). Interrogation of these networks at the level of transcripts, microbes, and phenotypes enables the study of mechanisms of microbiota influence on health and disease by identifying causal mechanisms responsible for phenotypic correlations.

**Figure 1:**
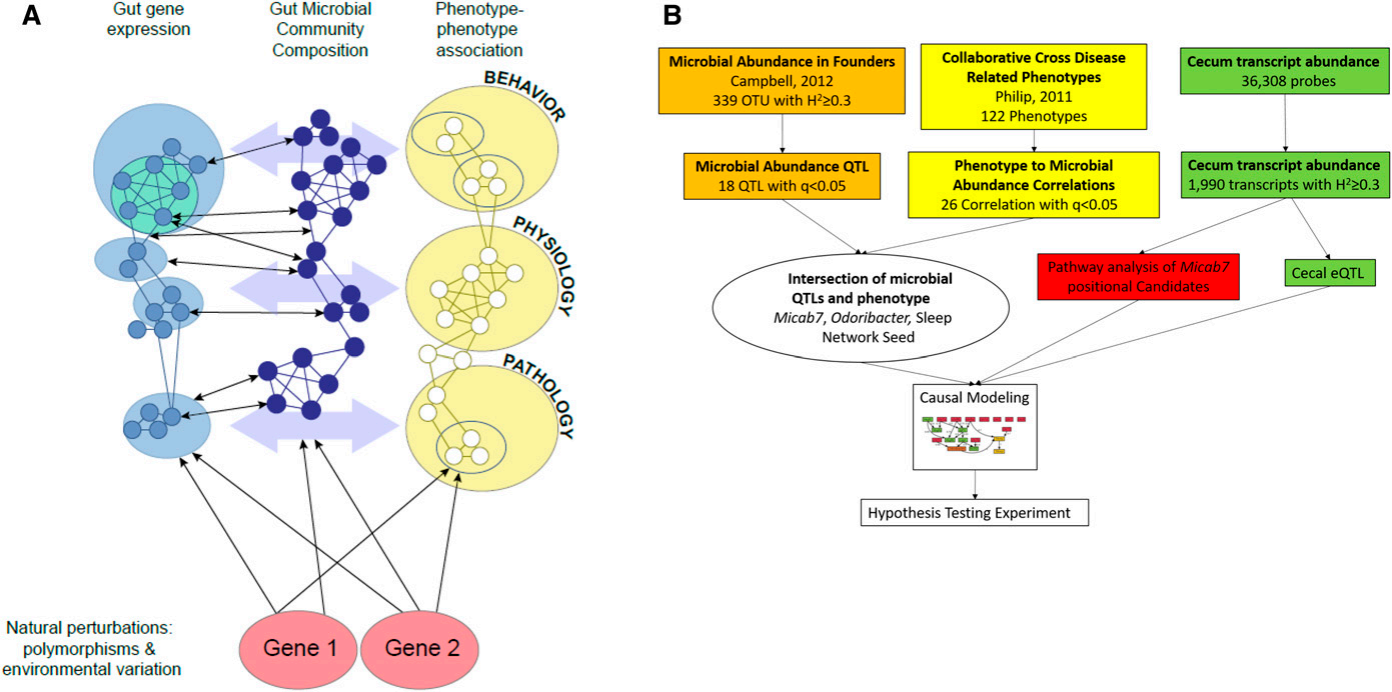
(A) The systems genetic model and gut microbiomics. Genetic variation in a host population together with the environment interact to affect intestinal gene expression in the host, microbial abundance, and disease-related traits in the host. There is significant bidirectional interplay among the microbiome, host gene expression, and disease-related phenotypes; however, the effect of host genotype is unidirectional, and therefore causal. (B) The analysis and decision steps used in this study of the relationship of the microbiome, cecal transcriptome, phenotype, and genotypes of Collaborative Cross mice, and then the analysis used in the validation experiment.

Here, we integrate data for host genotype, disease-related phenotypes, gut microbiome composition, and associated gut gene expression to develop a systems genetic network for the gut–microbiome interaction and its effects on host health. Specifically, we performed an integrative analysis leveraging cecal messenger RNA (mRNA) levels, luminal microbiome, physiology, and behavior from over 100 incipient strains of the CC population. Through the analysis of relationships among these measurements, we apply systems genetic analysis to identify a microbe involved in sleep disorders.

## Materials and Methods

### Mice

The breeding of the CC at Oak Ridge National Laboratory (ORNL) has been described previously (Chesler *et al.* 2008). Mice from each of the eight inbred progenitor strains consisting of five common inbred strains (A/J, C57BL/6J, 129S1/SvImJ, NOD/LtJ, and NZO/HILtJ) and three wild-derived inbred strains (CAST/EiJ, PWK/PhJ, and WSB/EiJ) were randomly assigned to one of a roughly balanced set of breeding schemes, which dictated the order in which strains were crossed. The strains were crossed pairwise to create a G1 generation, and these were crossed pairwise to make the four-way G2 generation, then crossed again to make the G2:F1 generation. The G2:F1s were crossed and the progeny randomly assigned to one of three mating pairs, of which one was randomly chosen as the priority pair to contribute to the next generation. If this mating was nonproductive, the offspring of the next ranked pair were used. To prevent die-out at advanced generations, backcrossing was also used in rare instances. A line was considered lost if no progeny were born after several breeding attempts using these strategies. Phenotyping and genotyping were performed in at least one breeding pair per line from generations G2:F5–G2:F8 for QTL analysis; most genotyped mice came from the G2:F5 generation, which we estimate to be 75% inbred (Philip *et al.* 2011).

All mice were housed at the William L. and Liane B. Russell Center for Comparative and Functional Genomics at ORNL. All animal protocols were reviewed and approved by the ORNL Institutional Animal Care and Use Committee (#0367; approval). They were maintained in separate cages either individually or with same-sex siblings, subjected to a light:dark cycle of (14:10), and allowed *ad libitum* access to the standard rodent chow (#5053; irradiated Purina Diet). Water was delivered via an automatic watering system chlorinated to 3–5 ppm. Cages contained Harlan Softcob bedding, with one nestlet enrichment device in each cage. Of the 650 CC lines initiated, 414 lines with at least a single male or female survived to the G2:F5 generation. Litters were weaned at ∼3 weeks of age into breeding pairs, until they entered the phenotyping protocol. Details of the CC Breeding population can be found in Philip *et al.* (2011).

BKS.Cg-*Dock7^m^* +/+ *Lepr^db^/J* mice (referred to here as db/db mice) were obtained from the Jackson Laboratory production colony, and used for the sleep and *Odoribacter* abundance experiments at The Jackson Laboratory. All animal protocols were reviewed and approved by The Jackson Laboratory Institutional Animal Care and Use Committee (#010007; approval).

### Phenotyping

When grandprogeny were born, members of the CC breeding population were subjected to high-throughput analysis broadly reflecting behavior, morphology, and physiology (Philip *et al.* 2011). Phenotypes included wildness, activity monitoring in open field, light/dark box, piezo sleep, hot plate, tail-clip, blood chemistry and cell counts, and fasting glucose. Dissection metrics of body weight and length, organ and fat pad weights, and bone composition were used. All CC phenotyping data and protocols are publicly available at the Mouse Phenome Database (phenome.jax.org; accession Chesler3).

### Dissection

Adult mice were euthanized with carbon dioxide and the cecum dissected. The number of animals varied such that for microbial abundance, 108 male and 108 female unique strains were used, and for genotyping 102 male and 99 female mice were used. Cecal contents were manually extruded and snap frozen. Each strain of mice was separately housed from the other strains, preventing any cohousing caging effects. The cecum tissue was extensively flushed with cold saline to get rid of residual fecal matter and snap frozen in RNAlater. All snap-frozen samples were stored at −80°.

### Extraction of microbial genomic DNA

DNA was extracted and the 16S ribosomal RNA (rRNA) gene amplified from cecum contents using a protocol modified from that of Ley *et al.* (2008), as previously described (Campbell *et al.* 2012). Approximately 100 mg of cecum contents was added to a 2-ml screw-capped tube containing 1 g of silica/zirconia beads (0.1 mm; BioSpec Products, Bartlesville, OK), 500 μl of phenol:chloroform:isoamyl alcohol (25:24:1), and 210 μl of 20% SDS. Headspace was filled with cold DNA extraction buffer (200 mM Tris at pH 8, 200 mM NaCl, and 20 mM EDTA). Bead tubes were attached to a vortex adapter (MO BIO, Carlsbad, CA) and shaken horizontally at high speed for 10 min. The aqueous phase was washed three times with phenol:chloroform:isoamyl alcohol (25:24:1) in phase gel lock tubes (QIAGEN, Valencia, CA). Nucleic acids were precipitated with 1 vol ammonium acetate (7.5 M) and 2 vol isopropanol, and incubation at −20° for ≥ 1 hr. Precipitated nucleic acids were concentrated by centrifugation at 15,000 × *g* for 15 min then dissolved in TE buffer. RNase A digestion (100 U) was performed for 30 min at 37°. Genomic DNA (gDNA) was precipitated with 0.1 vol sodium acetate (3 M, pH 5.5) and 3 vol ethanol, and incubation at −20° for ≥ 1 hr. Again, DNA was concentrated by centrifugation at 15,000 × *g* for 15 min, and pellets were washed twice with 70% ethanol, air dried, and dissolved in PCR-grade water. Mock extractions without cecum contents were used as negative controls.

### Preparation and pyrosequencing of the small subunit rRNA gene amplicon libraries in CC mice

Amplicon libraries of both V1–2 and V4 regions of the 16S small subunit (SSU) rRNA gene were obtained using protocols we described previously (Campbell *et al.* 2012). Amplification of the V1–2 region was performed in 50-μl reactions composed of 10X polymerase buffer (Invitrogen, Carlsbad, CA), 200 μM each dNTP, 3 mM MgSO_4_, 300 nM of forward primer (MWG Operon, Huntsville, AL), 300 nM reverse primer mix (MWG Operon), 1 U of Platinum Taq DNA Polymerase High Fidelity enzyme (Invitrogen), and 100 ng of gDNA. We used a modification of the 27F primer (Frank *et al.* 2008) fused to 6-nt multiplexing tags and to the 454-FLX sequencing primer A (5′-GCCTCCCTCGCGCCATCAGxxxxxx**GTTTGATCMTGGCTCAG**-3′), where the x region represents the multiplexing tag and the SSU rRNA primer is bold. A single reverse primer (5′- GCCTTGCCAGCCCGCTCAG**CTGCTGCCTYCCGTA**-3′) modified from Weisburg *et al.* (1991) was also used. Each amplification began with a denaturation step of 94° for 2 min followed by 25 amplification cycles of 94° for 20 sec, 53° for 30 sec, and 68° for 45 sec. A final extension at 68° for 3 min followed the amplification cycles. All amplicons were visualized on agarose gels for quality and subsequently purified from amplification reactions using Agencourt AMPure reagents (Beckman Coulter, Danvers, MA). A final check of amplicon quality and quantity was performed on an Agilent (Santa Clara, CA) Bioanalyzer using DNA 1000 reagents. Sequencing was performed on a 454-FLX instrument (Roche, Indianapolis, IN) following the manufacturer’s recommendations. Amplicon libraries V4 regions of 16S SSU rRNA gene were obtained using barcoded primers and sequenced using a 454-FLX instrument (Roche), using 40 samples per plate.

### Operational taxonomic unit-based sequence analysis

We utilized a taxonomy-independent analysis approach that classifies the sequences into operational taxonomic units (OTUs) based on sequence similarity (genetic distance), to avoid skewing the results with taxonomic relationships (Schloss and Handelsman 2004). High-throughput sequencing reads from the CC samples were clustered in the same analysis as the previously reported progenitor samples (Campbell *et al.* 2012). Reads were processed using the AmpliconNoise pipeline of Quince *et al.* (2011). The raw reads were filtered and trimmed using the underlying signal intensities or flowgrams generated by the 454 pyrosequencer. The first uncertain signal in each flowgram was found, *i.e.*, one with a value between 0.5 and 0.7, and the read truncated at this point. If this flow occurred prior to the 360th position in the 800 positions of the FLX Titanium read then it was discarded. In addition, all reads were truncated at the 720th flow. The flowgrams were then denoised using the PyroNoise step of the AmpliconNoise pipeline (Quince *et al.* 2009), which removes homopolymer errors. The flowgrams were then translated into sequences and further clustered by SeqNoise to eliminate PCR point errors, prior to chimera identification with Perseus. The filtered, denoised, and chimera-checked sequences were then clustered using a hierarchical clustering algorithm with average linkage and OTUs constructed at 3% sequence difference across all samples. Data were also analyzed with respect to taxonomic affiliation of the SSU rRNA gene fragments using the Ribosomal Database Project (RDP) Classifier set at an 80% confidence threshold. Counts of individual sequence hits to each annotated sequence cluster were obtained, providing a quantitative metric of relative microbial abundances at taxonomical classification levels that are comparable across human and mouse. Similarity of microbial profiles within and across mouse strains was evaluated, and host-specific microbial sequence clusters were identified.

### Heritability of microbial abundance

Intraclass correlation coefficients, which are used to estimate broad-sense heritability, were obtained from variance components attributable to progenitor mouse strain and residual error, based on data from a previous study in these strains (Campbell *et al.* 2012). The variance components were estimated using a linear mixed model including strain as a random effect. The strain intraclass correlation coefficients were calculated separately for females and males. The R/lme4 (http://www.r-project.org, R 3.1.2) package was used for these calculations (Supplemental Material, Table S1).

### Genotyping

A custom array using the Illumina iSelect platform for the Infinium system was developed for SNP genotyping as previously reported (Philip *et al.* 2011). Briefly, this array was based upon a subset of the 11,969 SNPs from the NIEHS-Perlegen SNP combined panel (Yang *et al.* 2007). The set was designed to discriminate all eight founder haplotypes and was optimized so that, for any SNP on the array, the maximum density of informative markers was used.

### mRNA preparation

Each dissected cecum was filled with a solution of 1.5 mM KCl, 96 mM NaCl, 27 mM sodium citrate, 8 mM KH_2_PO_4_, and 5.6 mM Na_2_HPO_4_ (pH 7.3) and incubated at 37°, after which the intestines were rinsed and filled with phosphate-buffered saline, 1.5 mM EDTA, and 0.5 mM dithiothreitol and incubated at 37° in a conical centrifuge tube. After an incubation time of 15 min, the tube was centrifuged at 900 × *g*. The resulting pellet was rinsed three times in phosphate-buffered saline (centrifuged at 900 × *g* for 5 min after each rinse). The resulting cell pellet was suspended in TRIzol (Invitrogen) and RNA was purified by column chromatography, as specified by the manufacturer’s protocol. Purified total RNA was fluorescently labeled with riboGreen (Invitrogen) and quantitated using a Spectamax Gemini XPS spectrofluorometer (Molecular Devices). RNA was then prepared for assay by dilution to 50 ng/µl in RNase-free water and transferred to a clean 1.5-ml assay tube.

### Whole-genome murine gene expression microarrays

All assay methods conformed to the exact protocol listed in the Whole-Genome Gene Expression Assay Manual. Diluted RNA (11 µl, ∼500 ng) was converted to biotinylated complementary RNA (cRNA) using the Illumina TotalPrep RNA Amplification Kit (Ambion). First, single-stranded cDNA was synthesized from the total RNA using the T7 Oligo (dT) primer, then converted to double-stranded cDNA using a combination of DNA polymerase and RNase H. Second, the cDNA was transcribed to biotinylated cRNA using the T7 enzyme and biotin-labeled NTPs. The resulting cRNA was column purified, quantitated, and diluted to 150 ng/µl in RNase-free water. The sample was then applied to a BeadChip (Illumina Mouse WG-6 v2 BeadChip) and hybridized overnight at 58° to allow the cRNA to anneal to the oligonucleotides corresponding to their specific gene. The BeadChips were then washed, blocked with E1, and fluorescently labeled with streptavidin-Cy3. BeadChips were dried by spinning at 300 rpm for 4 min in a benchtop centrifuge and imaged using the Illumina BeadArray Reader. Image data obtained from the BeadArray Reader were analyzed using BeadStudio version 3.0.19 with Gene Expression module 3.0.14. Rank invariant normalization was used with no background subtraction. We filtered the probes that targeted polymorphic sequences due to the bias introduced by microarray probe-target variation in genetic studies and the high density of mouse polymorphisms in the CC. The probes on the Illumina Mouse WG-6v2.0 BeadChip were tested by comparing the sequenced genomes from the Wellcome Sanger Institute (https://www.sanger.ac.uk/) (Yalcin *et al.* 2012) to determine if they contained a SNP between the strains. In total, 8972 probes were removed due to SNPs in one of the eight strains, and 36,308 probes remained for subsequent QTL mapping and genetic correlation analysis.

### Expression QTL mapping

For genetic mapping of gene expression, particularly *cis*-expression QTL (eQTL), large effects are detectable when the theoretical minor allele frequency (0.125 of the sample) is found in ≥20 mice (Valdar *et al.* 2006). To map the transcripts as individual traits, a SAS 9.1 (SAS Institute Inc., Cary, NC) heritability calculation was performed and probes/traits with heritability estimate (H^2^) < 0.30 were removed. This resulted in 1990 probes/traits to be mapped. Mapping of QTL was performed using a modified version of the DOQTL R package (Gatti *et al.* 2014). All genome scans were performed using the scanone() function in the DOQTL package with allele calls as input. A random effect was included to account for kinship effect. The *q*-values were calculated for each QTL to obtain multiple testing-adjusted *P*-values. An alternate model that used the CC mouse haplotypes and existing sequence data from the Sanger Mouse Genomes Project, and other sources, to infer individual mouse genotypes at all loci was used (Gatti *et al.* 2014). This enabled the application of genome-wide association to precisely identify those SNPs that predict phenotypic variation. The SNP association also improves precision, because the association is inferred directly at all polymorphic loci throughout the genome, rather than at specific typed SNPs, which may tag haplotypes with considerable numbers of linked polymorphisms. This latter approach improves statistical power because a single-SNP effect is estimated (one degree of freedom test) in contrast to the eight haplotype-specific effects that were used first.

### Mapping QTL for microbial abundance

In a genetic mapping and correlation study, genetic variation is randomized across the genome in the heterogeneous population, and each genotype is represented by multiple (∼1/8) individuals and segregates against a genetically randomized background. In this study, each mouse line resides in a distinct cage, further randomizing genetic and housing effects. Due to the large numbers of absent microbial OTU’s in individual mice, OTUs that were not present in ≥10% of the mice were removed, to ensure adequate power to detect correlations among traits and microbes. Application of this filtering step resulted in 846 OTUs in the incipient CC microbes data set. Each OTU was rank-Z-transformed and subjected to a genome-wide one-dimensional scan. The same methods were applied for the microbial QTL mapping as the eQTLs. The maximum LOD ratio of each OTU was recorded. Since the data were rank-Z-transformed, the first OTU was permuted and scanned 10,000 times to obtain genome-wide significance thresholds per trait. To account for the problem of multiple testing across the OTU’s, LOD scores were converted to an empirical *P*-value by calculating the proportion of permuted LOD scores found to be greater than the observed LOD score.

### Correlating OTU to behavior

Using previously reported phenotypes in the CC breeding population (Philip *et al.* 2011) we sought to correlate these with the microbial abundance of mice of the same line. We considered mice at generation G2:F5 and beyond. We performed missing value imputation for the behavioral phenotypes by replacing the missing values in G2:F5 with the next available value in the same breeding line, but with a higher generation. For example, if the phenotypic value for a certain mouse at G2:F5 was missing, we replaced this value by the value measured at G2:F6 or the value at G2:F7 if the value at G2:F6 was missing. Next, we removed all behavioral measures and OTUs containing only missing values or entirely zeros because these variables do not contribute to the genetic correlation analysis. There were a total of 205 animals in both data sets with 123 phenotypic measures and 13,618 OTUs. We computed the Kendall rank correlation coefficient (Kendall’s τ coefficient) for the behavioral phenotypes with OTUs using the cor.test() function in the R statistical framework (http://www.r-project.org/). Kendall’s τ is a nonparametric statistic that estimates the ordinal associations between two measures. The sign of the Kendall’s τ coefficient determines the direction of the relationship, while the magnitude of the correlation coefficient provides a measure of the strength of the relationship between behavioral phenotype and microbial abundance. Because of the sparse nature of the data, we handled the missing values by deleting all the cases with missing values. We hypothesized that there was no relationship between the behavioral phenotypes and the OTUs. The alternative was that there was a relationship in at least one behavioral phenotype and one of the OTUs. To investigate the relationship between the phenotypes and the OTUs, we performed the Student’s *t*-test implemented in the cor.test() function in R to test the hypothesis stated above. To account for the problem of multiple hypothesis testing, we applied the false discovery rate (FDR) adjustment implemented by the qvalue() function in the qvalue [R package version 1.38.0 (Storey *et al.* 2004)] package in R.

### Antibiotic treatment

All animal protocols were reviewed and approved by The Jackson Laboratory Institutional Animal Care and Use Committee (#010007; approval). Mice from a standard Specific Pathogen Free (SPF) colony were administered sulfatrim (19.75 mg/liter sulfamethoxazole + 3.95 mg/liter trimethoprim) and ampicillin (1 g/liter of ampicillin sodium salts, pharmaceutical grade) in their drinking water continuously from 8 weeks of age (Wu *et al.* 2010). The antibiotic exposure began in the breeding colony and continued into the testing phase. Trio matings of these mice were set up (db/+ × db/+) and the pups and lactating dames from these matings were given antibiotic water or control water continuously on a weekly basis. Sweetener (Equal) was added to the antibiotic and control water (2.5 g/liter). Male and female mice were used; sex was not significantly different and was collapsed across analysis. For sleep phenotyping, BKS.Cg-*Dock7^m^* +/+ *Lepr^db^/*J mice (referred to here as db/db mice) were used. Control db/db *n* = 5, and +/+ or db/+ *n* = 20. Antibiotic-treated db/db mice *n* = 24, and db/+ or +/+ *n* = 70.

### Preparation and sequencing of SSU rRNA gene amplicon libraries in mutant and treated mice

gDNA was extracted from all the samples using the PowerSoil DNA Isolation Kit. V1–3 regions of the 16S rRNA gene were amplified [27 F (5′-AGAGTTTGATCCTGGCTCAG-3′) and 534R (5′- ATTACCGCGGCTGCTGG-3′)], barcoded, and sequenced on the Miseq 2 × 300 bp sequencing platform. Data processing, including barcode removal, paired end assembly, quality trimming, and chimera screening were performed using a workflow scripted in Python (File S2). OTUs were generated using the Usearch package (version 8.0.1517), using the parameters indicated in this script. Taxonomical classification was based on RDP classifier 2.10, training set 11.

### Sleep phenotyping

Each mouse was placed in its own chamber atop a piezoelectric sensor for noninvasive sleep–wake scoring using PiezoSleep 1.0 (Signal Solutions, Lexington, KY) for 5-day sleep analysis (Flores *et al.* 2007; Donohue *et al.* 2008). The tester was always blind to genotype. The mice were randomly assigned to treatment or control groups where practical, and the mice had access to food and water *ad libitum* while in the chamber. The room was maintained on a 12:12-hr light:dark cycle. Mice were placed in the chambers between 9 and 10 am on day 1, and were removed on day 5 at the same time. The data acquisition computer, food, and water were checked daily; otherwise, the mice remained undisturbed. Measures recorded and analyzed consisted of activity onset, time of peak activity, sleep bout length, and total sleep time. The measure “% time sleep” was calculated for each hour and the 72 hr in the middle of testing, and was used for comparisons between groups. Statistical analyses were conducted using JMP 11 (SAS Institute). The best model is:

$$\%\;\mathrm{Sleep}=\beta_{0}\mathrm{Treatment+}\beta_{1}\mathrm{Genotype}+\beta_{2}{\left(\mathrm{Treatment}\times \mathrm{Genotype}\right)}_{+}\varepsilon$$

where ε is random error. The β-parameters were estimated by ordinary least squares and the type III sum of squares was considered for ε in the ANOVA model. In all cases, the full model was fit and reduced by dropping nonsignificant interactions followed by main effects.

To quantify the cyclic patterns in the sleep–wake behavior, a Fourier decomposition of the sleep percentage time series was computed to identify occurrences of < 24-hr sleep–wake cycles. Whereas the absolute gradient sum quantifies transitions between sleep–wake-dominated epochs, it does not distinguish between a single rapid change and many smaller ones over the period of interest. The Fourier amplitude is proportional to the cyclic changes between sleep- and wake-dominated epochs. Therefore, the maximum Fourier amplitude corresponding to cyclic activity with periods between 4 and 7 hr, *e.g.*, if no significant cyclic activity is present, is small (typically < 6). The full linear model was applied as above as well as a *post hoc* Tukey honest significant difference test for pairs of differences.

### Graphical modeling

Bayesian networks (BNs) are a subclass of directed probabilistic graphical models that were used to model the relationships between genotype and phenotype (Koller and Friedman 2009). Briefly, BNs depict the direct and indirect relationships between nodes in the network, and there is a direct relationship between the network topology and the joint distribution. Let *X* and Q be random variables representing the phenotypes and genotypes at SNP markers. Our objective was to learn the structure of the BN, which is an nondeterministic polynomial time (NP)-hard problem (Chickering *et al.* 2004). The local models were described using homogenous conditional Gaussian distributions, which allow for a mixture of discrete (genotypes) and continuous (phenotypes) variables (Lauritzen *et al.* 1989). We adopted the simplifying assumptions that SNPs are independent (unconnected) and that genotype precedes phenotype in the network structure. Briefly, the conditional distribution for a phenotype, $Y=X_{j}$, with discrete parent $Q_{i}$, with genotype states g, and continuous parents $X_{i}\left(i\neq j\right)$ can be expressed as:

$$P\left(Y\text{|}Q_{i}=g,X_{i}=x_{i}\right)=N\left(\alpha \left(g\right)+\beta {\left(g\right)}^{T}x_{i},\gamma \left(g\right)\right)$$

where the mean is a regression that depends on both the genotype states and continuous parent phenotypes, but the variance depends only on the genotype states. For count variables, the local models were described using a Poisson regression. The posterior distribution is given as:

P(G|D)αP(D|G)P(G)

where P(G) is the prior on the graph and P(D|G) is the likelihood. We used a noninformative energy prior embedded in a Gibbs distribution with hyperparamater τ=0.01. A Markov Chain Monte Carlo (MCMC) model was implemented to sample an ensemble of 1000 network structures from the posterior distribution (Hageman *et al.* 2011); the acceptance rate was 24%. Sex was considered a covariate for each local model. To preserve the data, samples with missing data were only eliminated from the affected local models and not from the global network. Model averaging was performed over the top 40 graphs, ranked by posterior probability, in the ensemble.

### Data availability

The microbial abundance of the progenitor strains is available at the Mouse Phenome Database [accession Chesler5 (RRID:nif-0000-03160)]. The gene expression microarray data are deposited at the Gene Expression Omnibus [(GEO:GSE96924) RRID:SCR_004584]. The genotypes and microbial abundances from the CC are available through the qtlarchive (RRID:nlx_151757) accession Bubier2, and the MsSeq of db/db animals at the National Center for Biotechnology Information Short Read Archive (SRA) [(PRJNA561132) RRID:SCR_004891]. All the QTL are deposited at the Mouse Genome Informatics database (RRID:nif-0000-00096) under accessions 5559705, 5559708, 5559710-5, 5559719-22, and 5559724-8. Figure S1, cecal microbial profile across mouse samples. Figure S2, eQTL map of cecum transcripts in CC mice. Figure S3A, microbes and sleep, and Figure S3B, antibiotic treatment and sleep. Figure S4, sleep phenotyping summary plot of sleep data from db/db antibiotic experiment. Table S1, founder strain intraclass correlations, sequences, and genus mapping based on the RDP. Table S2, heritability and eQTLs. Table S3, piezo sleep data from the db antibiotic study. Table S4, *post hoc* comparison of mean sleep fast Fourier transform (FFT) peak amplitude. Table S5, statistical analysis of sleep plots from Figure S4. File S1, denoised fasta sequences split by individual mice. File S2, commented command line for the MiSeq data analysis. Supplemental material available at figshare: https://doi.org/10.25386/genetics.11441550.

## Results

### Microbial community composition of incipient CC mice

The median broad-sense heritability of microbial abundance estimated by intraclass correlation in the CC founder strains data from Campbell *et al.* (2012) for each OTU (Table S1) was 0.170, with 339 OTUs having a H^2^ > 0.3, indicating sufficiently heritable abundance for genetic mapping. We determined the cecal microbial community composition of 206 CC mice of both sexes and 102 breeding lines using 454 pyrosequencing of amplicon libraries of the V4 region of the 16S SSU rRNA gene, revealing 13,632 OTUs. These samples were analyzed concurrently with the founder strains (Campbell *et al.* 2012) to obtain a single set of OTUs for both populations. Taxonomic analysis of all sequences using the RDP naïve Bayesian rRNA classifier (Cole *et al.* 2009, 2014) indicated bacterial diversity similar to that of previously observed communities (Campbell *et al.* 2012); Firmicutes comprised 89% of the microbial community and Bacteroidetes (9%) were the second most abundant phylum (Figure S1). In our previous study of replicate mice from the eight CC progenitor strains, we detected more phyla in the founders, but we show here that there are similar predominating phyla (Campbell *et al.* 2012) in the CC.

### Microbial abundance QTL

We performed QTL mapping to identify host genetic loci accounting for heritable variation in microbial abundance. There were 18 statistically significant (*q* < 0.05) microbial abundance (*Micab*) QTL (Table 1) among the mapped microbial OTU abundances. The 1.5 LOD C.I.s for the significant QTL range from 2 to 24 Mb in size, with an average size of 7.5 Mb. The size is consistent with previous mapping studies in the CC breeding and inbreed populations (Philip *et al.* 2011; Snijders *et al.* 2016), and substantially smaller than conventional experimental crosses. This interval size, coupled with the extensive genomic data becoming available for the CC founder population, enables refinement of the QTL down to the level of genes and variants in some cases.

**Table 1:** Significant QTL for microbial abundance in the cecum of incipient CC mice

MGI QTL symbol	QTL name	Chr	Peak LOD score	Peak marker	Position Mm 9 (bp)	1.5 LOD interval		P-value	Size (Mb)	Gene Weaver GSID
Micab1	Microbial abundance of Clostridiales Ruminococcaceae Oscillibacter 1	1	8.20	rs32084678	13,279,810	rs6275656	rs31653681	0.0033	6.34	217070
Micab2	Microbial abundance of Bacteroidales Porphyromonadaceae Paludibacter 2	3	8.23	rs31103355	108,854,325	rs31431100	rs37044521	0.0029	4.09	217071
Micab3	Microbial abundance of Clostridiales Lachnospiraceae Marvinbryantia 3	3	8.13	rs30089246	37,569,141	rs30552223	rs30158956	0.0037	2.03	217072
Micab4	Microbial abundance of Clostridiales Lachnospiraceae Roseburia 4	4	9.57	rs32690134	136,028,098	rs27619452	rs3685172	0.0005	9.45	217077
Micab5	Microbial abundance of Coriobacteriales Coriobacteriaceae Enterorhabdus 5	5	8.84	rs6377391	119,128,609	rs29633871	rs6354701	0.0009	4.65	217078
Micab6	Microbial abundance of Clostridiales Lachnospiraceae Sporobacterium 6	5	8.42	rs8265964	138,359,981	rs32246505	rs32318125	0.002	4.00	217079
Micab7	Microbial abundance of Bacteroidales Porphyromonadaceae Odoribacter 7	7	8.84	rs31494696	77,651,351	rs33107817	rs6373775	0.0009	3.53	217080
Micab8	Microbial abundance of Clostridiales Ruminococcaceae Lactonifactor 8	7	8.88	rs47611520	47,761,932	rs3661776	rs6176297	0.0009	13.55	217081
Micab9	Microbial abundance of Bacteroidales Porphyromonadaceae Odoribacter 9	7	8.60	rs31494696	77,651,351	rs33107817	rs6373775	0.0013	4.00	217082
Micab10	Microbial abundance of Clostridiales Lachnospiraceae Anaerostipes 10	8	9.61	rs32936112	47,123,375	rs6281843	rs31252778	0.0003	14.36	217083
Micab11	Microbial abundance of Clostridiales Incertae Sedis XIV Blautia 11	8	9.49	rs33429737	31,919,239	rs6399870	rs50110045	0.0005	5.19	217084
Micab12	Microbial abundance of Clostridiales Clostridiaceae Caminicella 12	9	8.29	rs30372085	80,440,479	rs30432532	rs33695839	0.0029	7.37	217092
Micab13	Microbial abundance of Erysipelotrichales Erysipelotrichaceae Turicibacter 13	10	8.87	rs29327022	88,018,183	rs6338556	rs6265280	0.0009	24.62	217093
Micab14	Microbial abundance of Bacteroidales Bacteroidaceae *Bacteroides* 14	11	8.14	rs26971743	58,783,410	rs6314621	rs26972849	0.0036	6.30	217094
Micab15	Microbial abundance of Clostridiales Lachnospiraceae Syntrophococcus 15	15	9.47	rs6388530	93,634,974	rs31931586	rs49819430	0.0005	10.43	217095
Micab16	Microbial abundance of Bacteroidales Porphyromonadaceae Tannerella 16	19	8.10	rs30320578	47,732,625	rs36280504	rs30760881	0.004	6.39	217096
Micab17	Microbial abundance of Clostridiales Ruminococcaceae Hydrogenoanaerobacterium 17	X	8.50	rs6292190	155,749,346	rs29276152	rs29306363	0.0017	5.59	217097
Micab18	Microbial abundance of Clostridiales Lachnospiraceae Lachnobacterium 18	X	8.20	rs6213950	163,790,061	rs8255374	rs31682358	0.0033	3.01	217098

Chr, chromosome; GSID, Gene Set ID; LOD, logarithm of the odds; MGI, Mouse Genome Informatics.

### eQTL in the CC cecum

To characterize the host intestinal state, we profiled mouse mRNA abundance in the cecal tissue surrounding the microbial sample. Transcript abundance estimates were generated for 36,308 microarray probes, representing 27,149 genes. Heritability of transcript abundance exceeded H^2^ = 0.3 for 1990 probes in the founder populations. QTL analysis was performed to identify host genomic regions harboring allelic variants that influence the abundance of each probe, resulting in the detection of statistically significant QTL (*q* < 0.05) for 1641 probes, corresponding to 1513 genes (Table S2). Of these, 950 loci (57.9%) were *cis*-eQTL (Figure S2), which contain polymorphisms that are proximal to transcript-coding regions. Such loci are useful in identifying expression regulatory mechanisms in the effects of genetic variation on complex traits.

### Genetic correlation of microbial abundance to disease-related traits reveals a microbe associated with sleep

Correlation of disease-related traits with underlying biomolecular and microbial characteristics across individuals provides a powerful means to identify previously unknown mechanisms of disease. A total of 122 disease-related behavioral and physiological phenotypes were correlated with the abundance of each OTU using Kendall’s τ, revealing 45 trait–microbe correlations (comparison-wise *P* < 0.05), 26 of which exceeded the multiple testing FDR threshold (*q* < 0.05) (Storey 2002) (Table 2). Of the trait–microbe pairs, 41 contained sleep phenotypes that showed significant comparison-wise Kendall’s τ correlations with 10 different microbes, 22 of which had *q* < 0.05. Among these, OTU 273 *Odoribacter* (order Bacteroidales, family Porphyromonadaceae) was the bacterium consistently correlated with the largest number of phenotypes (21 comparison-wise, 13 family-wise adjusted). Twelve of the family-wise significant correlations were with sleep phenotypes (Table 2 and Figure S3A).

**Table 2:** Correlations of disease-related phenotypes to microbial abundance

Phenotype	Kendall’s τ	*P*-value	*q*-value	Microbe (order, family, genus)		
Peak activity time from dark onset averaged over all baseline days (hr)	0.29	5.12E-06	0.036	Clostridiales	Lachnospiraceae	*Acetitomaculum*
Peak activity time from dark onset averaged over all baseline days (hr)	−0.29	7.44E-06	0.049	Bacteroidales	Bacteroidaceae	*Bacteroides*
Peak activity time from dark onset after sleep deprivation (hr)	−0.31	2.27E-06	0.017	Bacteroidales	Bacteroidaceae	*Bacteroides*
Average percentage of sleep time over all baseline days (%)	−0.30	5.24E-06	0.036	Bacteroidales	Porphyromonadaceae	*Barnesiella*
Glucose concentration (mmol/liter)	0.31	2.98E-06	0.022	Clostridiales	Lachnospiraceae	*Coprococcus*
Peak activity time from dark onset averaged over all baseline days (hr)	0.27	5.37E-06	0.037	Clostridiales	Lachnospiraceae	*Coprococcus*
Activity onset averaged over all baseline days (hr)	0.32	5.52E-07	0.008	Lactobacillales	Lactobacillaceae	*Lactobacillus*
Average of continuous sleep length over dark cycle in 4 full days (sec)	0.31	8.36E-07	0.009	Lactobacillales	Lactobacillaceae	*Lactobacillus*
Average of continuous sleep length over dark cycle for all baseline days (sec)	0.32	5.89E-07	0.008	Lactobacillales	Lactobacillaceae	*Lactobacillus*
Peak activity time from dark onset averaged over all baseline days (hr)	0.29	4.77E-06	0.034	Lactobacillales	Lactobacillaceae	*Lactobacillus*
Tail clip latency (sec)	0.31	1.02E-06	0.010	Lactobacillales	Lactobacillaceae	*Lactobacillus*
Creatinine concentration (mmol/liter)	0.47	8.26E-08	0.006	Clostridiales	Ruminococcaceae	*Lactonifactor*
Activity onset averaged over all baseline days (hr)	−0.29	2.55E-06	0.019	Bacteroidales	Porphyromonadaceae	*Odoribacter*
Average of continuous sleep lengths over the light cycle in 4 full days (sec)	−0.29	5.19E-06	0.036	Bacteroidales	Porphyromonadaceae	*Odoribacter*
Average of continuous sleep lengths over the light cycle for all baseline days (sec)	−0.28	5.61E-06	0.038	Bacteroidales	Porphyromonadaceae	*Odoribacter*
Average of continuous sleep lengths over the dark cycles in 4 full days (sec)	−0.31	1.13E-06	0.011	Bacteroidales	Porphyromonadaceae	*Odoribacter*
Average of continuous sleep lengths over the dark cycle for all baseline days (sec)	−0.30	1.90E-06	0.015	Bacteroidales	Porphyromonadaceae	*Odoribacter*
Average of continuous sleep lengths over 4 full days (sec)	−0.31	6.05E-07	0.008	Bacteroidales	Porphyromonadaceae	*Odoribacter*
Average of continuous sleep lengths over all baseline days (sec)	−0.30	1.41E-06	0.012	Bacteroidales	Porphyromonadaceae	*Odoribacter*
Peak activity time from dark onset averaged over all baseline days (hr)	−0.31	7.52E-07	0.009	Bacteroidales	Porphyromonadaceae	*Odoribacter*
Peak activity time from dark onset after sleep deprivation (hr)	−0.31	6.24E-07	0.008	Bacteroidales	Porphyromonadaceae	*Odoribacter*
Percentage of sleep over a 2-hr period prior to sleep deprivation (%)	−0.31	1.19E-06	0.011	Bacteroidales	Porphyromonadaceae	*Odoribacter*
Percentage of sleep time over the dark cycle of 4 full days (%)	−0.32	2.80E-07	0.007	Bacteroidales	Porphyromonadaceae	*Odoribacter*
Percentage of sleep time over 4 full days (%)	−0.33	1.22E-07	0.006	Bacteroidales	Porphyromonadaceae	*Odoribacter*
Tail clip latency (sec)	−0.29	3.91E-06	0.028	Bacteroidales	Porphyromonadaceae	*Odoribacter*
Average percentage of sleep time over all baseline days (%)	−0.30	6.44E-06	0.043	Clostridiales	Lachnospiraceae	*Roseburia*

### Genetic regulation of the abundance of Odoribacter

**Figure 2:**
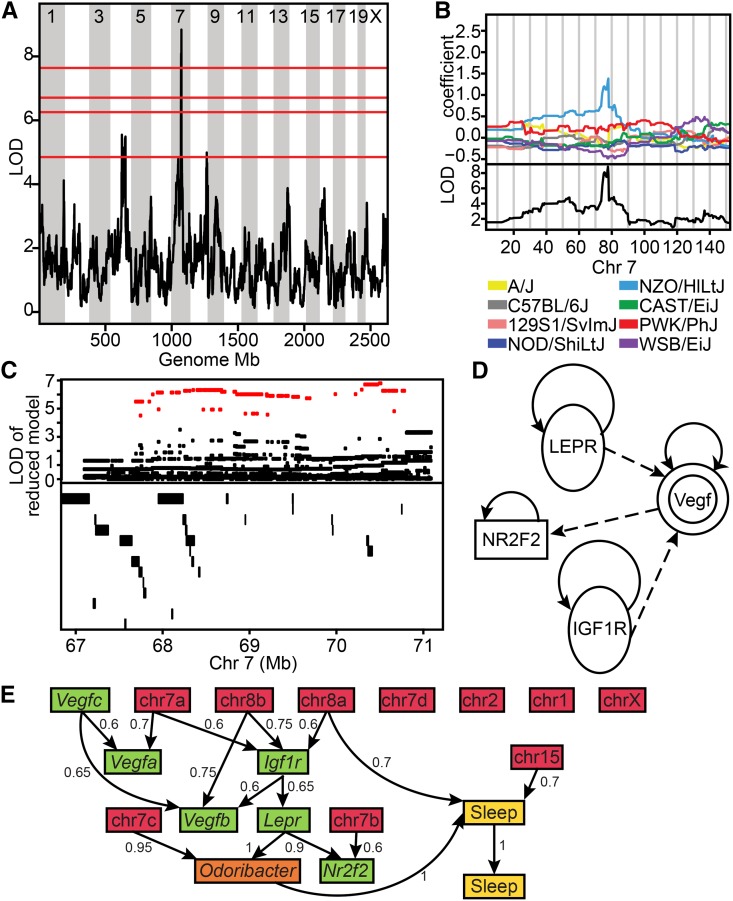
*Odoribacter* abundance in the cecum. (A) Genome scan showing a significant QTL (*P* < 0.01) peak on Chr 7. Horizontal lines represent permuted significance thresholds. From the top down: highly significant, *P* < 0.01; significant, *P* < 0.05; highly suggestive, *P* < 0.1; and suggestive, *P* < 0.63. (B) Detailed QTL map on chromosome 7. Bottom: LOD score across Chr 7. Top: allelic effect plots of eight coefficients of the QTL mixed model representing the effect of each CC founder haplotype on phenotype. The NZO allele on Chr 7 is associated with increased abundance of *Odoribacter* (C) Top: LOD score for SNP association mapping in the QTL support interval (67.1–71.1). Red points indicate SNPs with significant association to *Odoribacter* abundance. Bottom: genes and noncoding RNAs located in the QTL interval. (D) Ingenuity Pathway Analysis of the positional candidate genes together with *Lepr* show a network path through *Vegf*, and involving either *Nr2f2* or *Igf1r*. (E) Inferred network relating sleep, microbe abundance, microbial abundance QTL, expression correlations, and mutant mice. The network is a consensus representation of the 40 most likely Bayesian networks in an MCMC sample. Edge weights correspond to the marginal frequency of each directed edge in the top 40 BNs. Sleep 1 represents the sleep trait corresponding to the average of continuous sleep lengths over 4 full days, and Sleep 2 represents the sleep trait activity onset on the fourth day (hr). CC, Collaborative Cross; Chr, chromosome; MCMC, Markov Chain Monte Carlo.

The QTL *Micab7* on chromosome 7 is associated with the relative abundance of *Odoribacter*. The QTL is 3.53 Mb in size and contains 42 genes (Table 1). The allelic effects for each of the eight founder strain haplotypes are such that the NZO (New Zealand Obese) allele is associated with increased abundance (Figure 2, A–C). This is significant because the NZO founder strain is obese and prone to a diabetes phenotype, and previous studies of gut microbiota in obesity- and diabetes-prone mice revealed that *Odoribacter*, *Prevotella*, and *Rikenella* have been found in the microbiota of diabetic *db/db* (BKS.Cg-*Dock7^m^* +/+ *Lepr^db^*/J) mice, and are absent among db/+, +/+ littermates (Geurts *et al.* 2011). The *db/db* mice have also been shown to have abnormal sleep patterns in the form of altered sleep–wake regulation (Laposky *et al.* 2008).

We hypothesized that *Odoribacter*, *Lepr*, and sleep are connected through a common mechanism. Specifically, if the mechanism controlling altered sleep phenotype and the presence of *Odoribacter* in *Lepr* mutant *db/db* mice is the same mechanism that underlies the correlation of *Odoribacter* abundance and sleep in the CC mice, then we suspect that there is overlap between one or more of the QTL positional candidates and the *Lepr* pathway, and that the perturbation of the gut microbiota of *db/db* mice should affect sleep patterns.

To investigate whether there is overlap of *Micab7* QTL positional candidate genes and *Lepr*, we performed Ingenuity Pathway Analysis (IPA) on the 42 positional candidates, together with the gene *Lepr*. The most likely pathway from this database (Fisher’s exact test *P* < 10^−14^) contained the positional candidate genes *Nr2f2* and *Igf1r* interacting with *Lepr* through *Vegf* (Figure 2D).

Causal graphical models for phenotype–genotype networks (Rockman 2008) were used to infer the direct and indirect associations among the results of the IPA, including *Lepr*, *Vegfa*, *Vegfb*, *Vegfc*, the two positional candidates *Nr2f2* and *Igf1r*, the leptin pathway, and sleep. The network model included the cecal expression of the gene transcripts together with the abundance of *Odoribacter*, two sleep traits, and the genotypes of the CC mice at the QTL. BNs are described by directed acyclic graphs (DAGs), which can be efficiently decomposed and translated into the joint distribution of variables in the model (Koller and Friedman 2009). Conditional Gaussian distributions were used to model the relationships between genotype and phenotype, and the network structure was learned using an MCMC sampling scheme (Hageman *et al.* 2011) and averaging over the top structures (Hoeting *et al.* 1999). The graphical model was represented as a DAG, which could be efficiently decomposed and translated into the joint distribution of variables in the model. If a QTL was associated with the regulation of the *Vegf* pathway, we would expect to see evidence of a network edge between the genotype and at least one of the two positional candidates, the downstream *Lepr* genes, and the phenotype. Furthermore, this analysis can determine which positional candidate is most likely influenced by the causal variant. In aggregate summaries of the top 40 graphs, a repeatable relationship among the QTL, the positional candidate *Igf1r*, *Odoribacter*, and sleep was observed (Figure 2E). This relationship was observed in the majority of graphs. Therefore there is a plausible interaction among the QTL, *Igf1r* abundance, the leptin pathway, *Odoribacter*, and sleep.

### Broad-spectrum antibiotic treatment alters sleep patterns in Lepr^db^/Lepr^db^ mice

**Figure 3:**
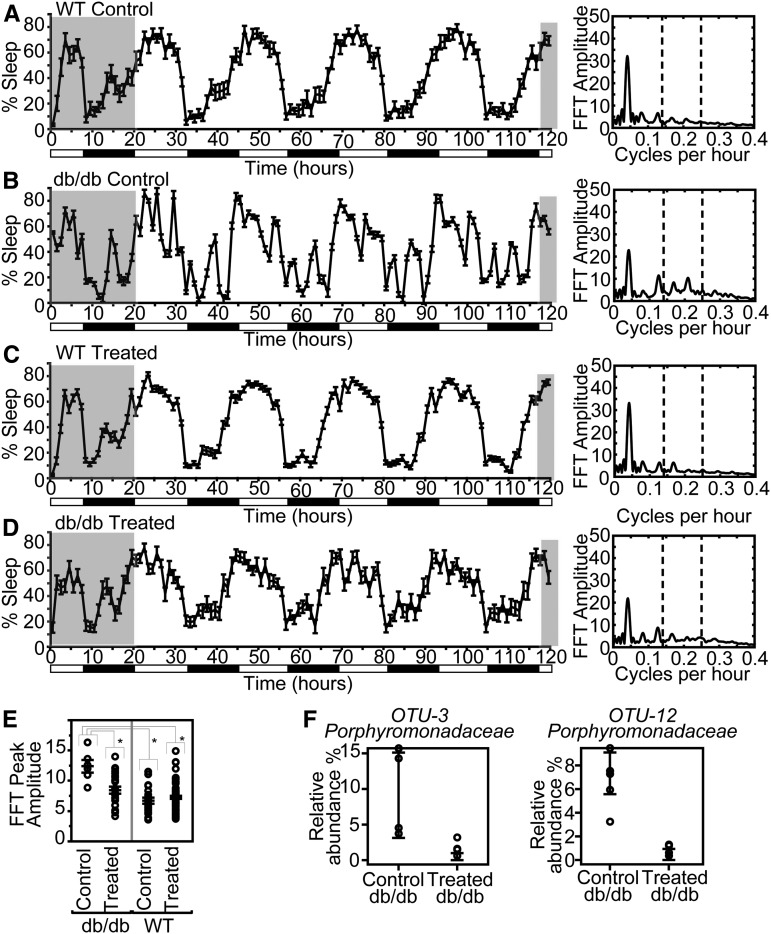
Mean and SE for the “% time sleep” over a 5-day test, with cyclic patterns characterized on the right by an FFT of the mean sleep percentage time series. (A) WT water only, (B) db/db mice water (C), WT given antibiotics, and (D) db/db mice given antibiotics. Genotype and antibiotic treatment have a significant interaction affecting “% Sleep” time. Night and day cycles shown with white and black bars, respectively. The FFT amplitudes of the regions of the % Sleep graphs in white are summarized in the graphs on the right. Percent sleep time for the antibiotic-treated *db/db* mice over a 72-hr period showed a genotype × treatment interaction in a repeated measure multivariate ANOVA; time × genotype × treatment F_(71,45)_ = 2.1199, *P* = 0.0040 (Figure 3, A–D). *Post hoc* contrast analysis revealed significant (*P* < 0.001) differences between control *db/db* mice and all three other groups (*P* < 0.001). (E) FFT peak amplitudes for each mouse corresponding to sleep percentage cycles, with periods ranging from 4 to 7 hr (shown with the dotted lines) during the final 4 days of the sleep cycle, shows significant genotype × treatment interaction as well as the db/db control being significantly different from the other three groups. (F) Two of the microbes present in the control db/db mice, but absent in the db/db antibiotic-treated mice. FFT, fast Fourier transform; WT, wild-type.

We then evaluated whether the presence of *Odoribacter* in *Lepr^db^* mice could explain the altered sleep behavior reported in these mice. To eliminate *Odoribacter*, mice were given antibiotic treatment continuously from conception. As expected (Savage and Dubos 1968), this broad-spectrum treatment resulted in increased fecal contents of the cecum observed at dissection in both genotypes (Figure S3B); however, it also resulted in a genotype-specific effect on sleep architecture. The percent sleep time for the antibiotic-treated *db/db* mice over a 72-hr period showed a genotype × treatment interaction in a repeated measure multivariate ANOVA: time × genotype × treatment F_(71,45)_ = 2.1199, *P* = 0.0040 (Figure 3, A–D), with *post hoc* contrast analysis showing significant (*P* < 0.001) differences between control *db/db* and all three other groups. The widely used sleep summary measurements, such as % time sleep in the light and dark phases, did not adequately capture the complexity of the differences between the control db and control wild-type strains, without or with antibiotics, as they did not reflect temporal patterns (rhythms) (Figure S4 and Table S5). There were genotype-specific differences between % sleep on day 1, day 2, day 3, and day 5 and in daily averages, which were not affected by treatment. Treatment appears to have had the greatest effect in a genotype-specific manner in the night (1, 2, 3, 4, and average), increasing the sleep time of db/db mice significantly. A Fourier amplitude analysis was performed using the FFT algorithm to identify cyclic patterns in sleep behavior. The amplitude of the Fourier spectrum reflects how dominant or consistent the cyclic pattern is at each frequency (cycles/hr) over the time period analyzed. All groups showed the most dominant peak at a period of 24 hr (∼0.042 cycles/hr) as expected. However, distinctions were seen in the peaks at higher frequencies (subcycles). In particular, strong differences were seen in the amplitudes between 0.14 and 0.25 cycles/hr (corresponding to periods ranging from 4 to 7 hr and highlighted by the vertical broken lines in Figure 3, A–D). Bout lengths will not necessarily impact these patterns. The peak heights quantify the presence of other (typically shorter) cycles relative to the main circadian cycle. Figure 3E shows the maximum amplitudes in the highlighted range for each mouse in the experimental groups. These show a significant genotype × treatment effect (F_(3,120)_ = 12.2193, *P* < 0.0001), and an individual least squares means Student’s *t*-test showed significant differences between control db/db mice and all three other groups (Table S4).

V4 sequencing of cecal contents from *db/db* mice showed seven microbial taxonomic units that were absent in the antibiotic-treated db/db case and elevated in the water vehicle (aspartame) control db/db, including two from the family containing *Odoribacter* (Figure 3F; SRA: PRJNA561132). Thus, the differential abundance of these seven microbial taxa, including *Odoribacter* abundance, is associated with genotype-specific effects of antibiotics on sleep architecture. It is possible that effects on other microbes, or other genotype-specific antibiotic effects, are responsible for the db/db-specific sleep pattern restoration; however, the systems genetic causal network analysis suggests that variation in *Odoribacter* abundance is the likely regulator of sleep architecture.

## Discussion

Using systems genetics and integrative functional genomics in the CC population, we traversed biological networks of genotypes, gene expression, microbes, and disease-related phenotypes to identify a host–microbe mechanism underlying sleep-related phenotypes. The high allelic variation and precision of the incipient CC mouse population allowed us to map loci that control the abundance of 18 particular microbes, which could be further decomposed using SNP analysis, haplotype association, and gene prioritization methods. Intestinal transcriptome profiling resulted in the detection of ∼1600 significant eQTL, and multiple clusters of transcripts and microbes, whose abundances are jointly modified by genetic variation. Through genetic correlation network analysis, we related these systems genetic networks to disease-related phenotypes obtained in the same population of mice. Causal network analysis and host genetic effects provided insight into the direction of host–microbe disease interactions.

Allelic variants influence the structure of microbial communities by creating conditions that promote or inhibit colonization by certain species (Spor *et al.* 2011). One way in which allelic variation manifests its effects is through the direct, or indirect, alteration of transcript abundance and the host environment, thereby impacting colonization. Other sources of variation may influence transcript abundance, including the presence of microbiota and their metabolites, disease states, and environmental variation. These sources of variation, and their association with microbiota and disease, can be detected through genetic correlation and probabilistic network analyses. By identifying network components and assessing causal relationships among them through experimental perturbation, it is possible to understand the mechanisms of these relationships.

Genetic correlation from mouse phenotype to microbial abundance enabled the identification of host and microbe influences on sleep architecture. The general role of microbes in sleep, particularly in the cytokine response to infection, is well documented (Krueger and Toth 1994). Previous work in rabbits (Toth and Krueger 1989) has shown that altered sleep patterns occur in response to an infectious challenge and that the sleep response is related to the type of infectious organism. Here, we report for the first time the relationship between the abundance of a specific microbe and sleep. OTU273 *Odoribacter* (Bacteroidales, Porphyromonadaceae) abundance is associated with the *Micab7* QTL and was correlated with multiple sleep phenotype measures. Genomic network analyses revealed that the primary candidate gene for the QTL is *Igf1r*, a gene likely to function in the regulation of sleep as the somatotropic axis, IGF-1 signaling, and sleep are intimately related (Obal *et al.* 2003). Perturbation of this pathway in the db/db *Lepr* mutant mouse is associated with abnormal phenotype and an elevated abundance of *Odoribacter*, albeit among other microbes. Both of these phenomena can be restored to normal values through antibiotic treatment. The observation that indigenous microbes could affect sleep patterns suggests the potential for probiotic or small-molecule metabolite development for the adjustment of sleep patterns in those with clinical sleep disorders. Other studies indicate a relationship between microbiota abundance and ultradian rhythms (Thaiss *et al.* 2014), and microbes of the *Odoribacter* were among five genera that decreased in feces in an intermittent hypoxia model of sleep apnea (Moreno-Indias *et al.* 2015).

The distinct changes to the sleep architecture and rhythms in, specifically, db/db mice in response to antibiotics are very interesting. The FFT shows the polyphasic *vs.* more monophasic sleep–wake pattern during the light and dark periods. This pattern is clearly visible and distinguishable by eye (Figure 3, A–D), and is best quantified with the FFT amplitude between 4 and 7 hr (in Figure 3E). This erratic pattern of sleep and wake may or may not be pathological, but it is clearly abnormal; however, we have not observed it in any of the common inbred strains or in wild mice that we have recently examined (Philip *et al.* 2011)

In these studies, we utilized systems genetic networks to identify, model, and validate the relationships among host genetics, genomics, microbiota, and disease. The mouse provides an efficient, well-controlled system in which to employ this approach, although it is amenable to application in human populations. We demonstrated that using mouse genetics, we can identify relationships that can be extrapolated to humans, though well-known issues in mechanistic conservation and direct translation must be considered. For example, despite high conservation across human and mouse genomes, specific biological mechanisms are not always entirely conserved, although the functional outputs of pathways and involvement in disease may be. Our approach to this challenge is to exploit network overlap, and to identify elements of mouse networks that can be translated to human genetic and genomic networks, which we expect to function similarly but perhaps differ in the details of specific allelic variants, genetic mechanisms, and particular microbiota involved. By developing our study around the holistic quantitation of both host and microbe, in contrast to typical studies of individual gene or treatment effects on microbiota, we are able to generate multiscale networks amenable to integration and extrapolation to disease mechanisms. Much remains to be done in the functional validation of the conservation of these mechanisms.

In all studies of the interplay between host environment, microbiota, and disease, the causal mechanisms underlying associations must be considered. Genetic variation influences the host environment, creating conditions that are hospitable or inhospitable ecological niches for gut microbiota, and can therefore be used to anchor a causal network. Identifying the precise causal genetic variants underlying microbial composition is a lengthy process that has become more tractable with deep sequencing of the CC founders, high-precision mapping populations including the Diversity Outbred derived from the CC, and the ability to integrate functional genomic data from other sources including epigenetic modification, noncoding variants, and disease associations. Although we have demonstrated that the QTL *Micab7* is associated with *Odoribacter* abundance, we have not yet demonstrated whether *Igf1r* variation is indeed the specific causal regulator of this phenotype, whether this locus is associated with abnormal *Odoribacter* abundance, and whether inoculation of *Lepr* or *Igf1r* mice with *Odoribacter* and its metabolites influences sleep. These extensive experimental manipulation studies will provide further evaluation of the causal network that we have identified. Alternatively the metabolites involved in this phenomenon may prove more feasible to identify and manipulate.

By exploiting genetic heterogeneity among organisms, we were able to extract mechanistic relationships between host, microbe, and disease. The systems genetic strategy employed herein provides a wealth of data resources that can be further interrogated by investigators with an interest in specific host genes, variants, microbes, and disease-related phenotypes. Furthermore, the strategy we present here can be readily deployed in other genetically diverse populations to provide efficient, holistic assessment of microbial and host mechanisms of disease. Extracting these disease-relevant mechanistic networks will provide insight into the complex interplay of host and microbe, revealing potential sources of disease etiology and points for therapeutic intervention.

## References

[bib1] Benson A. K., Kelly S. A., Legge R., Ma F., Low S. J., 2010 Individuality in gut microbiota composition is a complex polygenic trait shaped by multiple environmental and host genetic factors. Proc. Natl. Acad. Sci. USA 107: 18933–18938. 10.1073/pnas.1007028107 20937875 PMC2973891

[bib2] Bravo J. A., Forsythe P., Chew M. V., Escaravage E., Savignac H. M., 2011 Ingestion of Lactobacillus strain regulates emotional behavior and central GABA receptor expression in a mouse via the vagus nerve. Proc. Natl. Acad. Sci. USA 108: 16050–16055. 10.1073/pnas.1102999108 21876150 PMC3179073

[bib3] Campbell J. H., Foster C. M., Vishnivetskaya T., Campbell A. G., Yang Z. K., 2012 Host genetic and environmental effects on mouse intestinal microbiota. ISME J. 6: 2033–2044. 10.1038/ismej.2012.54 22695862 PMC3475380

[bib4] Carlisle E. M., Poroyko V., Caplan M. S., Alverdy J., Morowitz M. J., 2013 Murine gut microbiota and transcriptome are diet dependent. Ann. Surg. 257: 287–294. 10.1097/SLA.0b013e318262a6a6 23001074

[bib5] Carter C. J., 2013 Toxoplasmosis and polygenic disease susceptibility genes: extensive toxoplasma gondii host/pathogen interactome enrichment in nine psychiatric or neurological disorders. J. Pathogens 2013: 965046 10.1155/2013/965046 PMC3603208 23533776

[bib6] Chesler E. J., Miller D. R., Branstetter L. R., Galloway L. D., Jackson B. L., 2008 The Collaborative Cross at Oak Ridge National Laboratory: developing a powerful resource for systems genetics. Mamm. Genome. 19: 382–389. 10.1007/s00335-008-9135-8 18716833 PMC2745091

[bib7] Chickering D. M., Heckerman D., and Meek C., 2004 Large-sample learning of Bayesian networks is NP-hard. J. Mach. Learn. Res. 5: 1287–1330.

[bib8] Churchill G. A., Airey D. C., Allayee H., Angel J. M., Attie A. D., 2004 The Collaborative Cross, a community resource for the genetic analysis of complex traits. Nat. Genet. 36: 1133–1137. 10.1038/ng1104-1133 15514660

[bib9] Cole J. R., Wang Q., Cardenas E., Fish J., Chai B., 2009 The Ribosomal Database Project: improved alignments and new tools for rRNA analysis. Nucleic Acids Res. 37: D141–D145. 10.1093/nar/gkn879 19004872 PMC2686447

[bib10] Cole J. R., Wang Q., Fish J. A., Chai B., McGarrell D. M., 2014 Ribosomal Database Project: data and tools for high throughput rRNA analysis. Nucleic Acids Res. 42: D633–D642. 10.1093/nar/gkt1244 24288368 PMC3965039

[bib11] Deloris Alexander A., Orcutt R. P., Henry J. C., Baker J. Jr., Bissahoyo A. C., 2006 Quantitative PCR assays for mouse enteric flora reveal strain-dependent differences in composition that are influenced by the microenvironment. Mamm. Genome. 17: 1093–1104. 10.1007/s00335-006-0063-1 17091319

[bib12] Donohue K. D., Medonza D. C., Crane E. R., and O’Hara B. F., 2008 Assessment of a non-invasive high-throughput classifier for behaviours associated with sleep and wake in mice. Biomed. Eng. Online 7: 14 10.1186/1475-925X-7-14 18405376 PMC2365952

[bib13] Flores A. E., Flores J. E., Deshpande H., Picazo J. A., Xie X. S., 2007 Pattern recognition of sleep in rodents using piezoelectric signals generated by gross body movements. IEEE Trans. Biomed. Eng. 54: 225–233. 10.1109/TBME.2006.886938 17278579

[bib14] Frank J. A., Reich C. I., Sharma S., Weisbaum J. S., Wilson B. A., 2008 Critical evaluation of two primers commonly used for amplification of bacterial 16S rRNA genes. Appl. Environ. Microbiol. 74: 2461–2470. 10.1128/AEM.02272-07 18296538 PMC2293150

[bib15] Gatti D. M., Svenson K. L., Shabalin A., Wu L. Y., Valdar W., 2014 Quantitative trait locus mapping methods for diversity outbred mice. G3 (Bethesda) 4: 1623–1633. 10.1534/g3.114.013748 25237114 PMC4169154

[bib16] Geurts L., Lazarevic V., Derrien M., Everard A., Van Roye M., 2011 Altered gut microbiota and endocannabinoid system tone in obese and diabetic leptin-resistant mice: impact on apelin regulation in adipose tissue. Front. Microbiol. 2: 149 10.3389/fmicb.2011.00149 21808634 PMC3139240

[bib17] Goodrich J. K., Waters J. L., Poole A. C., Sutter J. L., Koren O., 2014 Human genetics shape the gut microbiome. Cell 159: 789–799. 10.1016/j.cell.2014.09.053 25417156 PMC4255478

[bib18] Hageman R. S., Leduc M. S., Korstanje R., Paigen B., and Churchill G. A., 2011 A Bayesian framework for inference of the genotype-phenotype map for segregating populations. Genetics 187: 1163–1170. 10.1534/genetics.110.123273 21242536 PMC3070524

[bib19] Hoeting J. A., Madigan D., Raftery A. E. and Volinsky C. T., 1999 Bayesian model averaging: a tutorial. Stat. Sci. 14: 382–401.

[bib20] Jacobs J. P., and Braun J., 2014 Immune and genetic gardening of the intestinal microbiome. FEBS Lett. 588: 4102–4111. 10.1016/j.febslet.2014.02.052 24613921 PMC4156569

[bib21] Khachatryan Z. A., Ktsoyan Z. A., Manukyan G. P., Kelly D., Ghazaryan K. A., 2008 Predominant role of host genetics in controlling the composition of gut microbiota. PLoS One 3: e3064 10.1371/journal.pone.0003064 18725973 PMC2516932

[bib22] Knights D., Silverberg M. S., Weersma R. K., Gevers D., Dijkstra G., 2014 Complex host genetics influence the microbiome in inflammatory bowel disease. Genome Med. 6: 107 10.1186/s13073-014-0107-1 25587358 PMC4292994

[bib23] Koller D., and Friedman N., 2009 Probabilistic graphical models: principles and techniques, MIT Press, Cambridge, MA.

[bib24] Krueger J. M., and Toth L. A., 1994 Cytokines as regulators of sleep. Ann. N. Y. Acad. Sci. 739: 299–310. 10.1111/j.1749-6632.1994.tb19832.x 7530430

[bib25] Krych L., Hansen C. H., Hansen A. K., van den Berg F. W., and Nielsen D. S., 2013 Quantitatively different, yet qualitatively alike: a meta-analysis of the mouse core gut microbiome with a view towards the human gut microbiome. PLoS One 8: e62578 10.1371/journal.pone.0062578 23658749 PMC3641060

[bib26] Laposky A. D., Bradley M. A., Williams D. L., Bass J., and Turek F. W., 2008 Sleep-wake regulation is altered in leptin-resistant (db/db) genetically obese and diabetic mice. Am. J. Physiol. Regul. Integr. Comp. Physiol. 295: R2059–R2066. 10.1152/ajpregu.00026.2008 18843095 PMC2685290

[bib27] Lauritzen S. L., Andersen A. H., Edwards D., Jöreskog K. G. and Johansen S., 1989 Mixed graphical association models. Scand. J. Stat. 16: 273–306.

[bib28] Leone V., Gibbons S. M., Martinez K., Hutchison A. L., Huang E. Y., 2015 Effects of diurnal variation of gut microbes and high-fat feeding on host circadian clock function and metabolism. Cell Host Microbe 17: 681–689. 10.1016/j.chom.2015.03.006 25891358 PMC4433408

[bib29] Lewin A. B., Storch E. A., Mutch P. J., and Murphy T. K., 2011 Neurocognitive functioning in youth with pediatric autoimmune neuropsychiatric disorders associated with streptococcus. J. Neuropsychiatry Clin. Neurosci. 23: 391–398. 10.1176/jnp.23.4.jnp391 22231309

[bib30] Ley R. E., Hamady M., Lozupone C., Turnbaugh P. J., Ramey R. R., 2008 Evolution of mammals and their gut microbes. Science 320: 1647–1651 (erratum: Science 322: 1188). 10.1126/science.1155725 18497261 PMC2649005

[bib31] Machiels K., Joossens M., Sabino J., De Preter V., Arijs I., 2014 A decrease of the butyrate-producing species Roseburia hominis and Faecalibacterium prausnitzii defines dysbiosis in patients with ulcerative colitis. Gut 63: 1275–1283. 10.1136/gutjnl-2013-304833 24021287

[bib32] McKnite A. M., Perez-Munoz M. E., Lu L., Williams E. G., Brewer S., 2012 Murine gut microbiota is defined by host genetics and modulates variation of metabolic traits. PLoS One 7: e39191 10.1371/journal.pone.0039191 22723961 PMC3377628

[bib33] Moreno-Indias I., Torres M., Montserrat J. M., Sanchez-Alcoholado L., Cardona F., 2015 Intermittent hypoxia alters gut microbiota diversity in a mouse model of sleep apnoea. Eur. Respir. J. 45: 1055–1065. 10.1183/09031936.00184314 25537565

[bib34] Obal F. Jr., Alt J., Taishi P., Gardi J., and Krueger J. M., 2003 Sleep in mice with nonfunctional growth hormone-releasing hormone receptors. Am. J. Physiol. Regul. Integr. Comp. Physiol. 284: R131–R139. 10.1152/ajpregu.00361.2002 12388430

[bib35] Parks B. W., Nam E., Org E., Kostem E., Norheim F., 2013 Genetic control of obesity and gut microbiota composition in response to high-fat, high-sucrose diet in mice. Cell Metab. 17: 141–152. 10.1016/j.cmet.2012.12.007 23312289 PMC3545283

[bib36] Philip V. M., Sokoloff G., Ackert-Bicknell C. L., Striz M., Branstetter L., 2011 Genetic analysis in the Collaborative Cross breeding population. Genome Res. 21: 1223–1238. 10.1101/gr.113886.110 21734011 PMC3149490

[bib37] Quince C., Lanzen A., Curtis T. P., Davenport R. J., Hall N., 2009 Accurate determination of microbial diversity from 454 pyrosequencing data. Nat. Methods 6: 639–641. 10.1038/nmeth.1361 19668203

[bib38] Quince C., Lanzen A., Davenport R. J., and Turnbaugh P. J., 2011 Removing noise from pyrosequenced amplicons. BMC Bioinformatics 12: 38 10.1186/1471-2105-12-38 21276213 PMC3045300

[bib39] Rockman M. V., 2008 Reverse engineering the genotype-phenotype map with natural genetic variation. Nature 456: 738–744. 10.1038/nature07633 19079051

[bib40] Savage D. C., and Dubos R., 1968 Alterations in the mouse cecum and its flora produced by antibacterial drugs. J. Exp. Med. 128: 97–110. 10.1084/jem.128.1.97 5662019 PMC2138511

[bib41] Schloss P. D., and Handelsman J., 2004 Status of the microbial census. Microbiol. Mol. Biol. Rev. 68: 686–691. 10.1128/MMBR.68.4.686-691.2004 15590780 PMC539005

[bib42] Snijders A. M., Langley S. A., Kim Y.-M., Brislawn C. J., Noecker C., 2016 Influence of early life exposure, host genetics and diet on the mouse gut microbiome and metabolome. Nat Microbiol. 2: 16221 10.1038/nmicrobiol.2016.221 27892936

[bib43] Spor A., Koren O., and Ley R., 2011 Unravelling the effects of the environment and host genotype on the gut microbiome. Nat. Rev. Microbiol. 9: 279–290. 10.1038/nrmicro2540 21407244

[bib44] Storey J. D., 2002 A direct approach to false discovery rates. J. R. Stat. Soc. Series B Stat. Methodol. 64: 479–498. 10.1111/1467-9868.00346

[bib45] Storey J. D., Taylor J. E. and Siegmund D., 2004 Strong control, conservative point estimation, and simultaneous conservative consistency of false discovery rates: a unified approach. J. R. Stat. Soc. Series B. Stat. Methodol. 66: 187–205. 10.1111/j.1467-9868.2004.00439.x

[bib46] Thaiss C. A., Zeevi D., Levy M., Zilberman-Schapira G., Suez J., 2014 Transkingdom control of microbiota diurnal oscillations promotes metabolic homeostasis. Cell 159: 514–529. 10.1016/j.cell.2014.09.048 25417104

[bib47] Toth L. A., and Krueger J. M., 1989 Effects of microbial challenge on sleep in rabbits. FASEB J. 3: 2062–2066. 10.1096/fasebj.3.9.2663582 2663582

[bib48] Turnbaugh P. J., Ley R. E., Mahowald M. A., Magrini V., Mardis E. R., 2006 An obesity-associated gut microbiome with increased capacity for energy harvest. Nature 444: 1027–1031. 10.1038/nature05414 17183312

[bib49] Turnbaugh P. J., Hamady M., Yatsunenko T., Cantarel B. L., Duncan A., 2009 A core gut microbiome in obese and lean twins. Nature 457: 480–484. 10.1038/nature07540 19043404 PMC2677729

[bib50] Valdar W., Flint J., and Mott R., 2006 Simulating the collaborative cross: power of quantitative trait loci detection and mapping resolution in large sets of recombinant inbred strains of mice. Genetics 172: 1783–1797. 10.1534/genetics.104.039313 16361245 PMC1456308

[bib51] Valles-Colomer M., Falony G., Darzi Y., Tigchelaar E. F., Wang J., 2019 The neuroactive potential of the human gut microbiota in quality of life and depression. Nat. Microbiol. 4: 623–632. 10.1038/s41564-018-0337-x 30718848

[bib52] Vijay-Kumar M., Aitken J. D., Carvalho F. A., Cullender T. C., Mwangi S., 2010 Metabolic syndrome and altered gut microbiota in mice lacking Toll-like receptor 5. Science 328: 228–231. 10.1126/science.1179721 20203013 PMC4714868

[bib53] Weisburg W. G., Barns S. M., Pelletier D. A., and Lane D. J., 1991 16S ribosomal DNA amplification for phylogenetic study. J. Bacteriol. 173: 697–703. 10.1128/JB.173.2.697-703.1991 1987160 PMC207061

[bib54] Wen L., Ley R. E., Volchkov P. Y., Stranges P. B., Avanesyan L., 2008 Innate immunity and intestinal microbiota in the development of Type 1 diabetes. Nature 455: 1109–1113. 10.1038/nature07336 18806780 PMC2574766

[bib55] Willing B. P., Dicksved J., Halfvarson J., Andersson A. F., Lucio M., 2010 A pyrosequencing study in twins shows that gastrointestinal microbial profiles vary with inflammatory bowel disease phenotypes. Gastroenterology 139: 1844–1854.e1. 10.1053/j.gastro.2010.08.049 20816835

[bib56] Wu H. J., Ivanov I. I., Darce J., Hattori K., Shima T., 2010 Gut-residing segmented filamentous bacteria drive autoimmune arthritis via T helper 17 cells. Immunity 32: 815–827. 10.1016/j.immuni.2010.06.001 20620945 PMC2904693

[bib57] Yalcin B., Adams D. J., Flint J. and Keane T. M., 2012 Next-generation sequencing of experimental mouse strains. Mamm. Genome. 23: 490–498. 10.1007/s00335-012-9402-6 22772437 PMC3463794

[bib58] Yang H., Bell T. A., Churchill G. A., and Pardo-Manuel de Villena F., 2007 On the subspecific origin of the laboratory mouse. Nat. Genet. 39: 1100–1107. 10.1038/ng2087 17660819

